# Black tea preserves intestinal homeostasis through balancing barriers and microbiota in mice

**DOI:** 10.3389/fnut.2024.1367047

**Published:** 2024-05-21

**Authors:** Yuxuan Shi, Shasha Guo, Jihong Zhou, Ping Xu, Yuefei Wang

**Affiliations:** ^1^Institute of Tea Science, Zhejiang University, Hangzhou, China; ^2^Key Laboratory of Horticultural Plant Growth, Development and Quality Improvement, Ministry of Agriculture, Hangzhou, China

**Keywords:** black tea, intestinal homeostasis, intestinal barrier, gut microbiota, inflammation

## Abstract

**Introduction:**

Black tea, a beverage consumed worldwide, possesses favorable effects on gastrointestinal tract, including nourishing stomach and promoting digestion. Nevertheless, its specific effects on intestinal homeostasis remains inconclusive.

**Methods:**

We applied black tea to mice prior to inducing colitis with DSS and then monitored their body weight and disease activity index (DAI) daily. When sacrificed, we measured intestinal permeability and conducted analyses of mucin and tight junction proteins. We detected inflammatory cytokines, immune cells, and related inflammatory signaling pathways. In addition, the gut microbiota was analyzed through 16S rRNA sequencing, and the concentrations of short-chain fatty acids (SCFAs) were also measured.

**Results:**

The results showed that black tea-treated group significantly rescued the DSS-disrupted intestinal structure. It reduced the relative abundance of the pathogenic bacterium *Turicibacter*, while increased the abundance of beneficial bacteria *norank_f_Muribaculaceae* and restored the contents of SCFAs such as acetate, propionate, and butyrate. It also protected the intestinal barrier by reducing the levels of immune response-related factors (e.g., TNF-α, IL-6, IL-1β) and increasing the expression of tight junction proteins (TJs) (e.g., ZO-1, occludin). Furthermore, black tea exhibited the capacity to suppress the expression of *MMP-9* and *ICAM-1*, as well as to inhibit the activation of NF-κB signaling pathway.

**Discussion:**

Our findings provide a theoretical framework that elucidates the mechanisms by which black tea preserves intestinal homeostasis, highlighting its potential as a preventive strategy against intestinal disruptions. This study contributes to the understanding of the dietary effects of black tea on gastrointestinal health.

## 1 Introduction

Intestinal homeostasis is a dynamic balance in intestine and tightly regulated by such as intestinal mucosal barrier, intestinal environment (including intestinal microbiota and their respective metabolites) (1). Among them, intestinal mucosal barriers can be classified into mechanical, chemical, immune and biological barriers according to their functions (2). In recent years, biological barriers consisting of resident microorganisms in gut have become a hot research topic. Gut microbiota has been shown to enhance the stability of intestinal barrier and to play an immunomodulatory role inside and outside of gut (3). At the same time, its metabolites (e.g., SCFAs and bacterial indole metabolites, etc.) are supposed to contribute to strengthening the mucosal barrier and attenuating the inflammatory response. Yang et al. (4) has elucidated the mechanism by which SCFAs promotes IL-22 production by immune cells, thereby maintaining the stability of intestinal internal environment. It suggests that SCFAs have a positive and important impact on the “immune-metabolic-flora” interactions in intestinal homeostasis (5, 6). Damage to the intestinal mucosa can trigger a variety of gastrointestinal disorders such as inflammatory bowel disease and even intestinal tumors (7). In addition, gut and gut microbiota establish a communication network with other organs through neural, endocrine, immunological, humoral and metabolic pathways, influencing other organs and triggering associated diseases (8). Thus, maintaining intestinal homeostasis is critical in the prevention and treatment of various diseases.

As the primary interacting factor with gut microbiota, food intake directly and significantly impacts intestinal homeostasis. Scientific research has validated the wisdom behind the Chinese concept of “the same origin of medicine and food,” which highlights correlations between healthy dietary habits and reduced risks of metabolic diseases (9). On the contrary, unhealthy diets have been identified as crucial contributors to the degradation of epithelial gut barrier integrity in mammals (10, 11). Tea consumption is widely regarded as a health-promoting habit (12). Enormous evidence has shown that tea and its contained bioactive compounds have positive effect on health, including inhibition of cancer cells (13), anti-inflammatory attributes (14), cardiovascular protection (15), enhancement of liver function (16), blood sugar regulation (17, 18), neuroprotective qualities (19), promotion of skin health (20, 21), and favorable effects on bone health (22), whereas their specific action mechanisms are still unclear. Recent research has underscored the gut microbiota may play a pivotal role in the health benefits of tea (23, 24). For example, the application of tea polysaccharides conjugates (TPS) in Ulcerative colitis (UC) mice significantly altered the composition of the gut microbiome, contributing to the restoration of intestinal homeostasis (25). Another study demonstrated that green tea polyphenols can alleviate the symptoms of acute colitis and suppress the development of inflammation-related colon cancer through the regulation of intestinal microorganism composition and metabolite levels (26). Nevertheless, the aforementioned studies primarily focused on the partial active compounds in tea, while in typical daily consumption, the total intake of tea constitutes the majority. However, our comprehension of the impact of this routine daily consumption pattern on intestinal homeostasis remains partial, thus restricting the potential utility of tea across a range of physiological circumstances.

Here, black tea, the most globally consumed tea, was used as the primary research material to explore the mechanisms by which tea consumption habits influence gastrointestinal health. We established a DSS-induced intestinal damage mouse model of gut homeostasis imbalance and evaluated the effects of pre-supplementing with black tea on intestinal damage. Specifically, our study delves into how black tea pre-treatment contributes to maintaining gut microbiota balance and restoring physical, chemical, and immune barriers in the intestine. These findings support the notion that black tea is a beneficial beverage in alleviating intestinal inflammation, and demonstrate the positive impact of regular tea intake on health.

## 2 Materials and methods

### 2.1 Materials and chemicals

“Longjing 43” tea leaves produced in Hangzhou, Zhejiang Province, China were processed into black tea. Preparation included a 12-h withering period, 1 h rolling, 3 h fermentation at 30°C and 3 h drying at 90°C. DSS was procured from MP Biomedicals (Mw 36–50 kDa, Santa Ana, United States). Enzyme linked immunosorbent assay (ELISA) kits for interleukin 6 (IL-6), interleukin-1β (IL-1β), tumor necrosis factor-α (TNF-α), Myeloperoxidase (MPO), zonula occludens-1 (ZO-1), and Occludin were obtained from Solarbio (Beijing, China). Standards of acetic acid, propionic acid, butyric acid, isobutyric acid, pentanoic acid, and valeric acid were purchased from Aladdin Reagent Co., Ltd. (Shanghai, China).

### 2.2 Preparation of tea infusion and dosage determination

The black tea infusion were prepared at two concentrations, low and high, based on the standard daily tea consumption for a 60 kg human (2 g of black tea brewed with 150 mL of boiling water, with an overall daily intake of 1,200–2,000 mL (27)). Human tea consumption metrics were recalibrated for mice through the Meeh-Rubner formula. The resulting tea solution concentrations for a 20 g mouse were established as 8.2 mg/mL for the low dose (BTL) and 16.4 mg/mL for the high dose (BTH). For the preparation, 1.64 g and 3.28 g of black tea were each brewed with 200 mL of sterile water at 100°C. After steeping for 10 min, the tea leaves were filtered out, and the resulting black tea solution was collected in the mice’s drinking bottles.

### 2.3 Composition of black tea

Gallic acid (GA) was used as a standard to determine the total phenol content according to GB/T 8313–2018 (ISO 14502-1:2005). Neutral sugar content was determined by anthrone-sulfate acid method using D-glucose as the standard (28); protein content was analyzed by Bradford method (29) using bovine serum albumin as the standard; free amino acids were determined by ninhydrin colorimetric method (GB/T 8314–2013). Theabrownins (TBs) and thearubigins (TRs) was measured by the spectrophotometry method according to NY/T 3675–2020, theaflavins (TFs) was determined by high performance liquid chromatography (HPLC) according to GB/T 30483–2013. In simple terms, 0.2 g of tea leaves are extracted in a 70°C water bath for 10 min, then cooled to room temperature. The mixture is centrifuged at 3500 rpm for 10 min, the supernatant collected, and the extraction repeated. Extracts are combined to a volume of 10 mL and filtered through a 0.45 μm membrane. Chromatographic analysis uses a C18 column with mobile phase A (30 mL acetonitrile, 5 mL acetic acid, 965 mL water) and phase B (300 mL acetonitrile, 5 mL acetic acid, 695 mL water). A gradient elution of 30–85-30% B phase is applied over 40–60-68 min, with a column temperature of 50°C, detection wavelengths at 250 nm and UV360 nm, a flow rate of 1 mL/min, and an injection volume of 10 μL.

### 2.4 Animal experimental design

**Figure 1:**
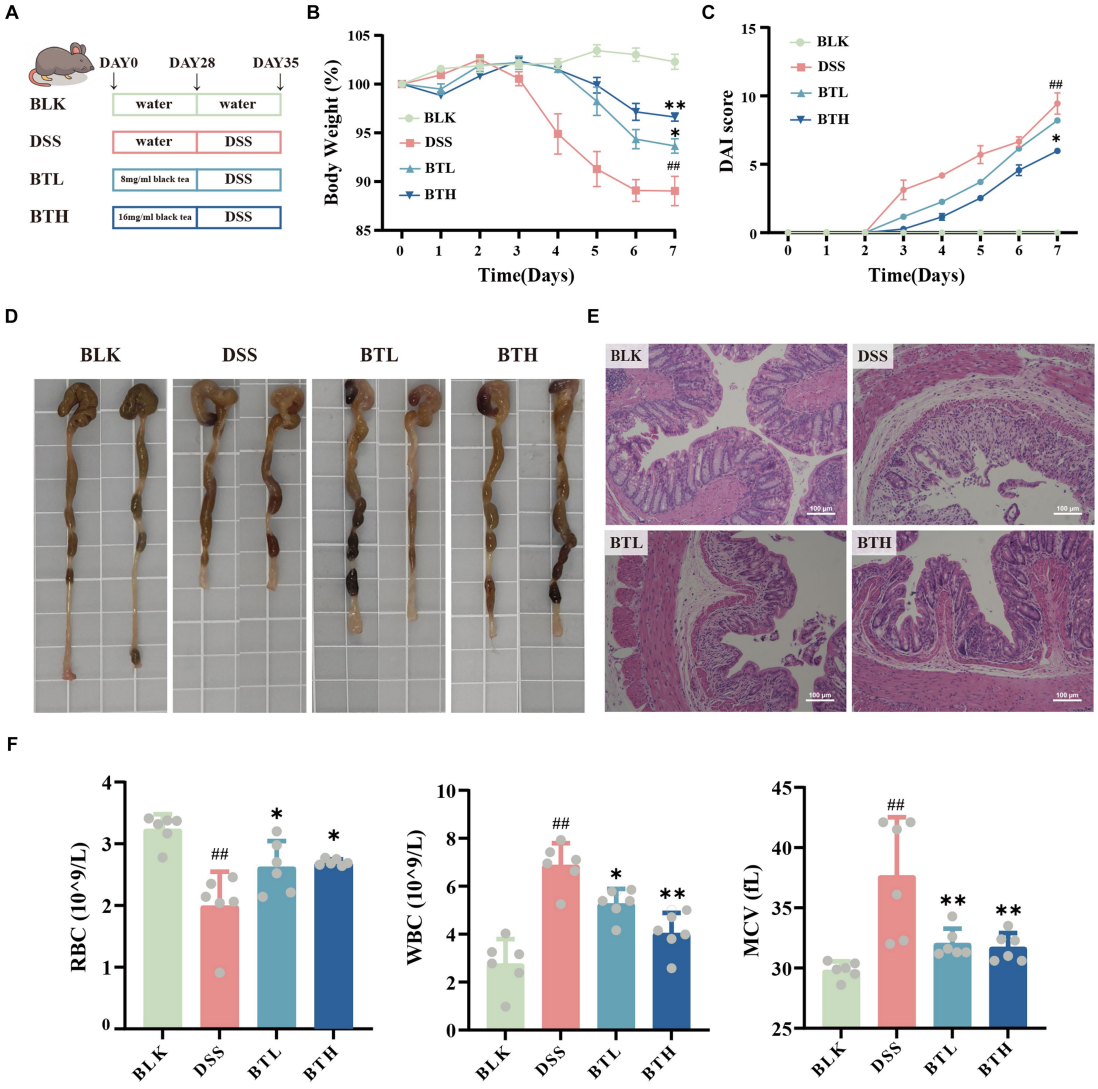
Effects of black tea preventive action on the clinical symptoms of DSS-induced colitis in mice. **(A)** Schematic diagram of experimental design. **(B)** Body weight changes. **(C)** Disease Activity Index (DAI). **(D)** Images of the colon. **(E)** Colonic H&E staining (×400). **(F)** Hematological analysis: RBC, WBC, MCV. Data are presented as mean ± SEM (represented by error bars; *n* = 6). The significance of differences between groups was calculated using one-way analysis of variance and Tukey’s multiple comparison test. Compared to the BLK group, ^#^*p* < 0.05, ^##^*p* < 0.01; compared to the DSS group, ^*^p < 0.05, ^**^p < 0.01.

Male C57BL/6 J mice (4-week-old, 18 ± 2.0 g) were purchased from Shanghai SLAC Laboratory Animal Co. Ltd. (SLAC, China), with a production license number SCXK 2017–0005. All mice were housed in the SPF clean environment at the Laboratory Animal Centre of Zhejiang University, using the animal license NO.SYXK (Zhe) 2022–0037. The light/dark cycle was 12 h, relative humidity was 55–60%, and temperature was 23–26°C. After 1 week of acclimatization, the mice were randomly divided into four groups (8 mice per group): BLK, DSS, BTL, and BTH. BLK and DSS groups were administered sterile water, BTL was given 8 mg/mL, and BTH was given 16 mg/mL black tea without additional water, which was changed every day to maintain freshness. After 4 weeks, acute colitis was induced with 2.5% DSS for 1 week, except for the control group, which received only sterile water (Figure 1A). All mice had unrestricted access to standard laboratory diet, water or tea. Daily measurements included mouse weight, water intake and food intake. All animal experiments were approved by the Experimental Animal Ethics Committee of Zhejiang University (ZJU20190004).

### 2.5 Sampling procedure

During the induction of acute colitis, animals were observed daily for changes in body weight, fecal characteristics and occult blood and scored according to established criteria. These scores were combined to calculate the Disease Activity Index (DAI) for each animal. At the end of the experiment, all mice were humanely executed. Mouse serum, colon, cecum and spleen were collected for subsequent analysis. Serum samples were allowed to stand for 2 h, then centrifuged at 3,000 rpm for 20 min and stored at −80°C. The serum samples were then analyzed using an automated analyzer. Mouse alanine aminotransferase (ALT) and blood urea nitrogen (BUN) were measured using an automated analyzer. The entire colon was resected, washed with phosphate buffer solution (PBS) and its contents collected. The tissue was blotted dry and the length of the colon was measured. The splenic tissue was dissected and weighed to calculate the splenic index. Collect the colonic tissue and store 1 cm distal to it in 4% paraformaldehyde. The remaining colon tissue was dissected longitudinally according to the manufacturer’s instructions and subjected to RNA extraction and ELISA to assess levels of IL-6, IL-1β, TNF-α, MPO, Occludin, and ZO-1.

### 2.6 Histological characterization of colon tissue

The distal colon tissue from mice was fixed in 4% paraformaldehyde for 24 h, paraffin-embedded, and subsequently subjected to Hematoxylin and Eosin (H&E) staining. The Histological Damage Index (HI) was evaluated in accordance with established protocols. The tissue sections were then examined under a microscope for observation and image capture.

### 2.7 Quantitative analysis of gene expression in colon tissue

Total RNA was extracted from colon tissue using an RNA extraction kit (Vazyme Biotech). The quantity and purity of total RNA were assessed using a NanoDrop 2000 spectrophotometer (Thermo Fisher Scientific Inc., Waltham, MA, United States) as per the manufacturer’s instructions. Total RNA was reverse transcribed to cDNA using PrimeScript reverse transcription master mix (TaKaRa Bio). Quantitative real-time PCR (qRT-PCR) was performed using SYBR Green Master Mix and the Quant Studio 6 Flex Real-Time PCR System according to the instructions. The expression levels of relevant genes were calculated by the 2^−ΔΔCt^ method using GAPDH as internal reference. The primer sequences are shown in Supplementary Table S1.

### 2.8 Immunohistochemical analysis of tight junction protein

Paraffin sections of mouse colon tissue were fixed with 4% paraformaldehyde and then deparaffinized with xylene and alcohol solution. Antigen recovery was performed after heating in citrate buffer. After blocking non-specific antigens with goat serum, primary antibodies (anti-ZO-1, Occludin) were used and incubated at 4°C overnight. Horseradish peroxidase-labeled secondary antibody was then added, followed by DAB color development, hematoxylin counterstaining, running water back to blue, gradient ethanol solution and xylene dehydration, and coverslip sealing. More than three random regions of each sample were imaged under a 40 × objective. Target proteins were analyzed semi-quantitatively using Image J software, and the intensity of protein expression was indicated by the area covered by positive proteins.

### 2.9 16S rDNA sequencing and gut microbiota analysis

Total DNA was extracted using the e.z. n.a.^®^ Soil Kit (Omega Bio-Tek, Norcross, GA, United States) and the contents of the blind bladders were subjected to high-throughput 16S rDNA sequencing. The V3-V4 region was amplified using primers 338F and 806R. PCR products were purified, quantified by QuantiFluor™-ST (Promega, United States) and pooled for sequencing on the Illumina MiSeq platform. Raw sequences were processed for quality control, splicing and noise reduction to obtain amplicon sequence variants (ASVs). ASVs were classified using the Naive bayes classifier in Qiime2 based on the Silva 16S rRNA gene database (v138). Data analysis was performed on the Majorbio cloud platform.^1^

### 2.10 Determination of SCFAs

Acetic acid (40 mg/mL), propionic acid (16 mg/mL), butyric acid, isobutyric acid, isovaleric acid and valeric acid (3.2 mg/mL each) were prepared in a 5 mL volumetric flask with water to form a standard stock solution. After mixing and addition of hydrochloric acid, the mixture was processed and filtered before injection. Chromatographic conditions consisted of the mobile phases acetonitrile and 25 mmol/L potassium dihydrogen phosphate buffer (pH 2.3) in a ratio of 14: 86 (v/v). The flow rate was 1.0 mL/min, the detection wavelength was 210 nm, the column temperature was maintained at 45°C, and the injection volume was 20 μL. The chromatographic conditions were as follows.

### 2.11 statistical analysis

Results were expressed as mean ± SEM. Differences between groups were analyzed by one-way analysis of variance (ANOVA) followed by Tukey’s *post hoc* test. *p*-values <0.05 were considered statistically significant.

## 3 Results

### 3.1 Pretreatment with black tea alleviated the clinical symptoms of DSS-induced colitis in mice

In an attempt to explore the potential prophylactic role of black tea in preserving gut homeostasis, colitis was induced in C57BL/6 mice utilizing DSS. To determine whether black tea intervention affected water intake in DSS-induced colitis mice, daily water consumption was meticulously monitored. The black tea prophylaxis did not influence the water consumption in the DSS-induced colitis mice (Supplementary Figure S2A). Seven days post DSS treatment, mice exhibited significant (*p* < 0.01) weight loss, emergence of colitis syndromes like diarrhea or bloody stools, a stark increase in the disease activity index, and a conspicuous enlargement of the spleen (Supplementary Figure S3A). Conversely, the black tea prophylaxis groups, BTL and BTH, showed lesser weight loss, and a slower escalation in the DAI compared to the DSS group (Figures 1B,C). Additionally, a dose-dependent amelioration of spleen index was observed (Supplementary Figure S3B). The control group displayed normal colonic tissue appearance and thickness, devoid of congestion, edema, or adhesion with the surrounding tissues. The DSS-treated mice evidenced significant (*p* < 0.05) shortening of the colon, accompanied by congestion, which was relieved by black tea intervention, mitigating the DSS-induced reduction in colon length (Figure 1D). To further assess the pathological changes in the colon, Hematoxylin–Eosin (HE) staining was carried out. The DSS group demonstrated anomalies in crypt structures and epithelial morphology, specifically, damage to the mucosal layers on the intestinal epithelial cell surface, severe glandular destruction leading to crypt disappearance, and extensive inflammatory cell infiltration, As revealed in Figure 1E. The BTL group, while displaying certain degrees of crypt upwards migration, irregular surface structures, and cryptic distortion, managed to retain the fundamental structures. The black tea prophylaxis improved the severe epithelial damage and crypt deformation triggered by DSS in a dose-dependent manner. Compared with the BLK group, red blood cells (RBC) and mean corpuscular hemoglobin contentration (MCHC) were significantly reduced (*p* < 0.05) and mean corpuscular volume (MCV) was significantly increased (*p* < 0.05) in the model group, indicating significant anemia in mice with DSS-induced colitis. In contrast, BTL and BTH group showed an increase in RBC counts and a decrease in white blood cells (WBC) counts. Concurrently, DSS-induced colitis mice exhibited ALT and BUN activities markedly higher than their normal counterparts, but Black tea prophylaxis can reverse this trend (Supplementary Figures S2E,F). This suggests that long-term consumption of black tea can effectively alleviate clinical symptoms and complications in DSS-induced colitis mice.

### 3.2 Pretreatment with black tea regulated the gut microbiota imbalance and improved SCFA metabolism of DSS-induced colitis in mice

**Figure 2:**
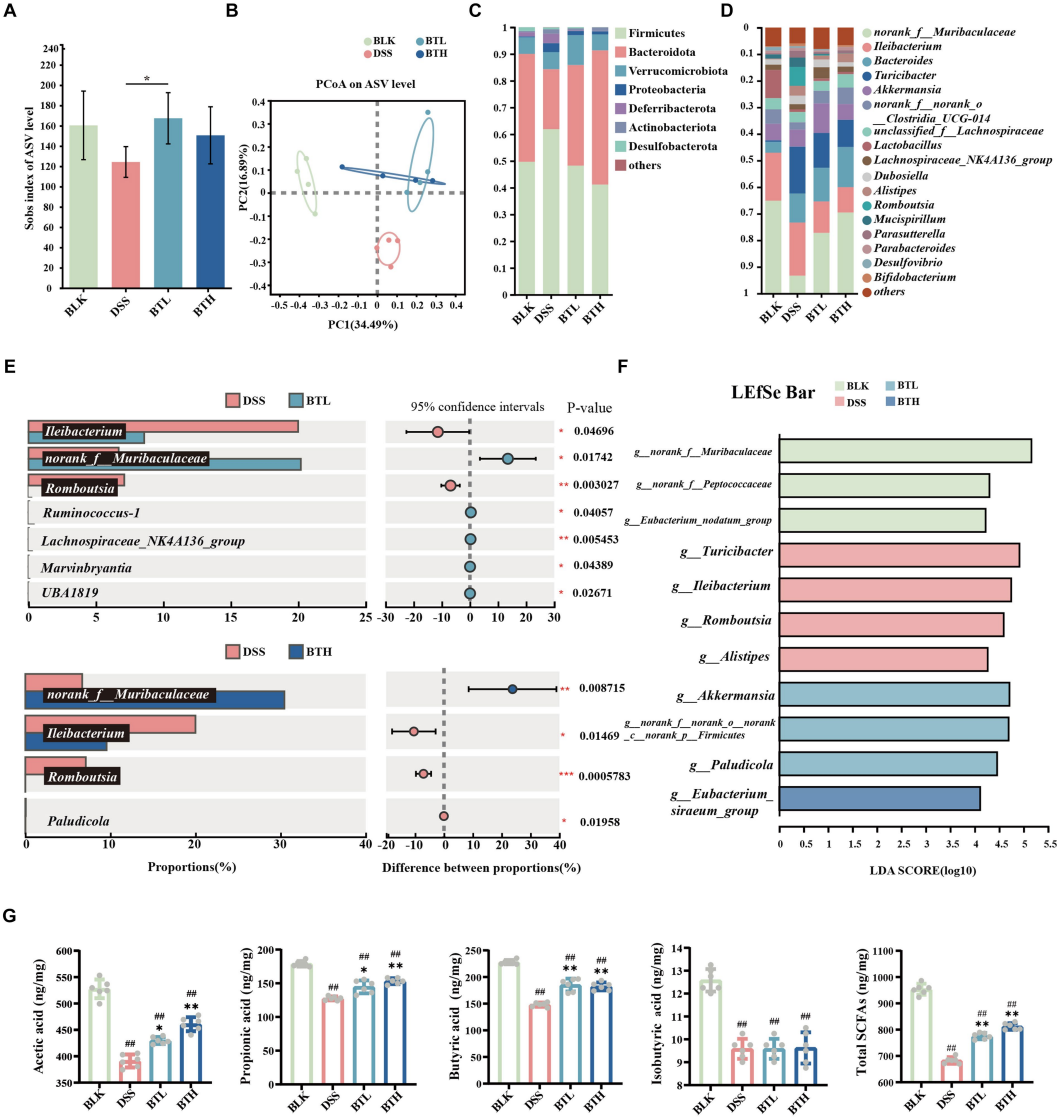
Black tea prevention regulated gut microbiota and promoted the production of SCFAs. **(A)** Alpha diversity analysis using the Sobs index. **(B)** PCoA analysis. **(C)** Composition of microbial communities at the phylum level. **(D)** Composition of microbial communities at the genus level. **(E)** Student’s *t*-test bar plot on genus level (Comparison of DSS with BTL, DSS with BTH). **(F)** The results of the LEfSe analysis are shown in a bar graph of linear discriminant analysis (LDA > 2, *p* < 0.05). **(G)** Contents of acetic acid, propionic acid, butyric acid, isobutyric acid, and total SCFAs. Data are presented as mean ± SEM. The significance of differences between groups was calculated using one-way analysis of variance and Tukey’s multiple comparison test. Compared to the BLK group, ^#^*p* < 0.05, ^##^*p* < 0.01; compared to the DSS group, ^*^p < 0.05, ^**^p < 0.01.

To investigate changes in gut microbes, an analysis of colon fecal bacterial composition was conducted in four groups of mice through 16S rRNA gene sequencing. The Sobs index at the ASV level represents microbiota richness. Under the DSS condition, this richness declined, while the black tea preventative group reinstated it. Moreover, there was a statistically significant difference between DSS and BTL (*P* < 0.05) (Figure 2A). In addition, PCoA was utilized to explore the gut microbiota composition among different groups (Figure 2B), where PC1 explained 34.49% of the total data variation. The separation between DSS and BLK suggested a significant difference in their microbial community compositions. While there was minimal difference between the black tea preventative groups BTL and BTH, their confidence intervals were distinct from DSS, implying that black tea intervention triggered significant changes in gut microbiota structure. At the phylum level (Figure 2C). Relative to BLK, DSS increased the abundance of Firmicutes (*p* < 0.05), Bacteroidetes, Proteobacteria, and Actinobacteria, and decreased Bacteroidetes abundance (*p* < 0.05). In black tea-pretreated colitis mice, a distinct microbiota composition was identified when compared to DSS, where BTH displayed a lower Firmicutes abundance (*p <* 0.001) and a higher Bacteroidetes content (*p* < 0.001).

At the dominant genus level (Figure 2D), *norank_f_Muribaculaceae*, *Ileibacterium*, and *Bacteroides* were identified as the dominant microbial taxa in the mouse gut microbiota across all groups. Comparative analysis of species differences between the two groups revealed significant (*p* < 0.01) downregulation of *norank_f_Muribaculaceae*, *Lactobacillus*, *Eubacterium_ventriosum_group*, and *Anaerotruncus* in the DSS group compared to the BLK group. Conversely, there was a significant (*p* < 0.01) upregulation of *Bacteroides*, *Turicibacter*, *Romboutsia*, *unclassified_f_Anaerovoracaceae* in the DSS group. Following pretreatment with high and low concentrations of black tea, the relative abundance of *norank_f_Muribaculaceae* and *Ileibacterium* increased, while the abundance of *Turicibacter* and *Romboutsia* decreased. Notably, BTL exhibited significant upregulation of *Ruminococcus-1*, *Lachnospiraceae_NK4A136_group*, *Marvinbryantia*, and *UBA1819*, whereas BTH exhibited significant downregulation of Paludicola (*p* < 0.05).

As depicted in Figure 2F, the LEFSe analysis revealed an enrichment of *Turicibacter*, *Ileibacterium*, *Romboutsia*, and *g_Alistipes* in the DSS group. At the BTL group, there was a significant enrichment of *Akkermansia*, *norank_f_norank_o_norank_c_norank_p_Firmicutes*, and *Paludicola* (*p* < 0.05). In the BTH group, *g_Eubacterium_siraeum_group* was the dominant genus. Further analysis of SCFAs in the intestines of mice revealed that, compared to the BLK group, the total amount of SCFAs in the DSS group significantly decreased (*p* < 0.01). The black tea prevention groups improved the overall SCFA levels by increasing the amounts of acetate acid (10% increase in BTL compared to the DSS control group, and 18% increase in BTH compared to the DSS control group), propionate acid (13% increase in BTL compared to the DSS control group, and 20% increase in BTH compared to the DSS control group), and butyrate acid (25% increase in BTL compared to the DSS control group, and 23% increase in BTH compared to the DSS control group). Although DSS reduced the total SCFA levels by 28%, the treatment groups elevated the total SCFA levels to near those of the BLK group (Figure 2G), constituting a significant difference (*p* < 0.05).

### 3.3 Pretreatment with black tea enhanced the gut mechanical and chemical barrier of DSS-induced colitis in mice

**Figure 3:**
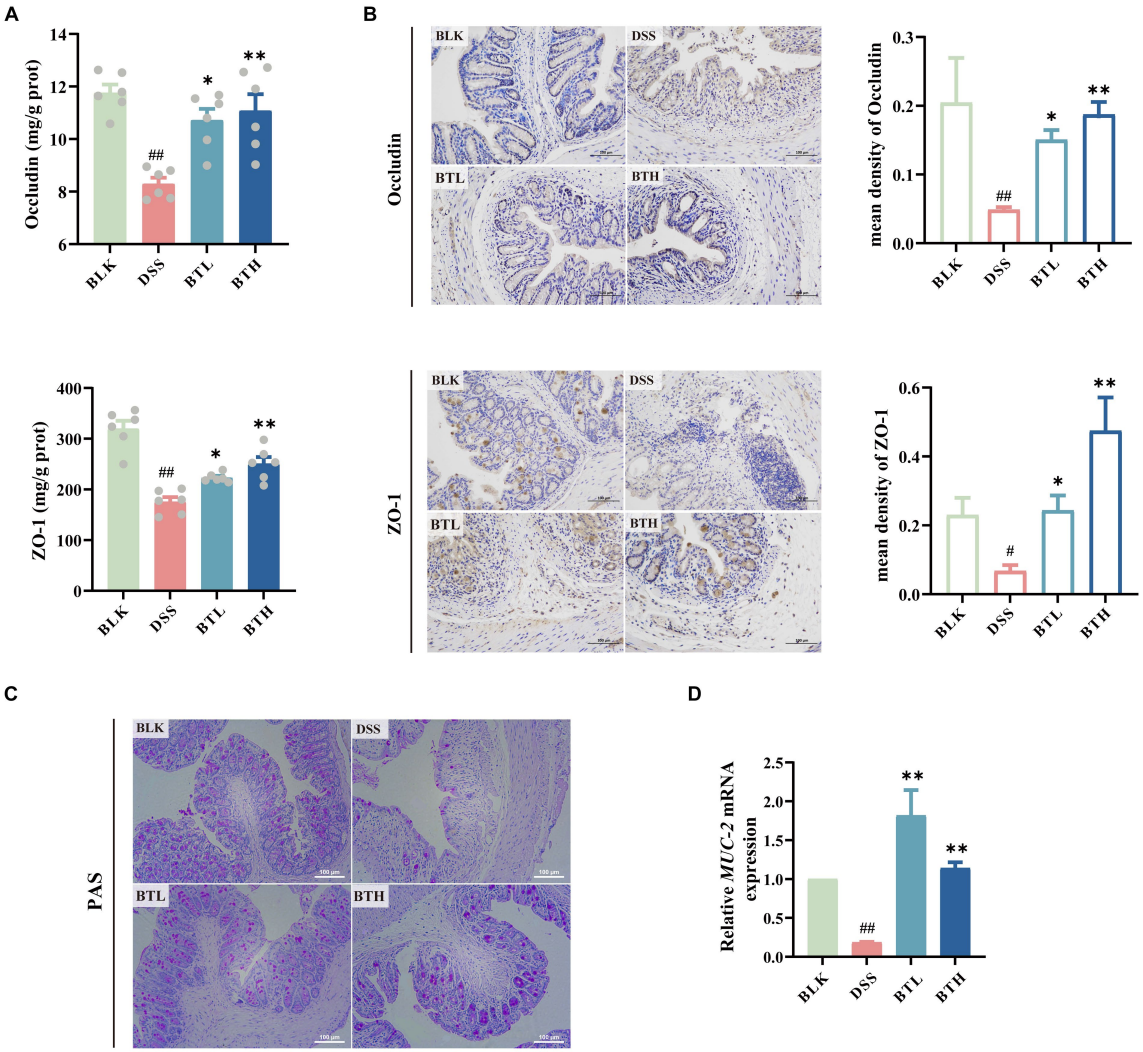
Protective effects of different doses of black tea prevention on the mechanical and chemical barriers of the gut in DSS-induced mouse colitis. **(A)** Elisa detection of tight junction protein levels Occludin, ZO-1 (*n* = 6); **(B)** Immunohistochemical detection of colonic tight junction protein expression levels and Average optical density values of Occludin and ZO-1 (×200); **(C)** colonic PAS staining (×400); **(D)** qRT-PCR detection of mucin (*MUC-2*) genes in colonic tissues (*n* = 3). Data are presented as mean ± SEM (represented by error bars). The significance of differences between groups was calculated using one-way analysis of variance and Tukey’s multiple comparison test. Compared to the BLK group, ^#^*p* < 0.05, ^##^*p* < 0.01; compared to the DSS group, ^*^p < 0.05, ^**^p < 0.01.

Both ELISA and immunohistochemical methods were employed to assess the expression levels of TJs, including ZO-1 and occludin. The protein concentration of ZO-1 and occludin in the colon of DSS-exposed mice was considerably lower compared to the mice in the normal BLK group (*p* < 0.01) (Figure 3A). Interestingly, mice in the black tea pre-treatment group exhibited an augmented expression of ZO-1 and occludin proteins. Consistent results were further provided by the immunohistochemical experiment, where the mean optical density (MOD) of ZO-1 and occludin was quantified using image analysis software. A distinct decrease in brownish granules of occludin and ZO-1 in the DSS group was observed, along with an increase in light areas, indicative of a lower protein expression level in this region. Remarkably, black tea pre-treatment enhanced the positive staining and significantly improved (*p* < 0.01) the content of TJs in a dose-dependent manner (Figure 3B), thereby alleviating intestinal mucosal damage instigated by impaired tight junctions. In addition, the mucin in the goblet cells of the mouse colon tissue was stained purple red using the PAS staining technique (Figure 3C). A noteworthy decline in goblet cells’ abundance was detected in the DSS group compared to the normal group, along with a near disappearance of the mucin layer. Conversely, a marked increase in the mucin layer and goblet cell distribution was witnessed in the BTL and BTH groups. Employing the qRT-PCR method, we detected the relative expression of Mucin 2 (Muc-2) mRNA. In comparison to the BLK group, the Muc-2 mRNA level in the DSS group’s colon showed a significant decrease (*p* < 0.01) (Figure 3D). However, black tea pre-treatment groups significantly (*p* < 0.01) increased in the Muc-2 mRNA expression level compared to DSS group, and even surpassed the BLK group.

### 3.4 Pretreatment with black tea strengthened the gut immune barrier of DSS-induced colitis in mice

**Figure 4:**
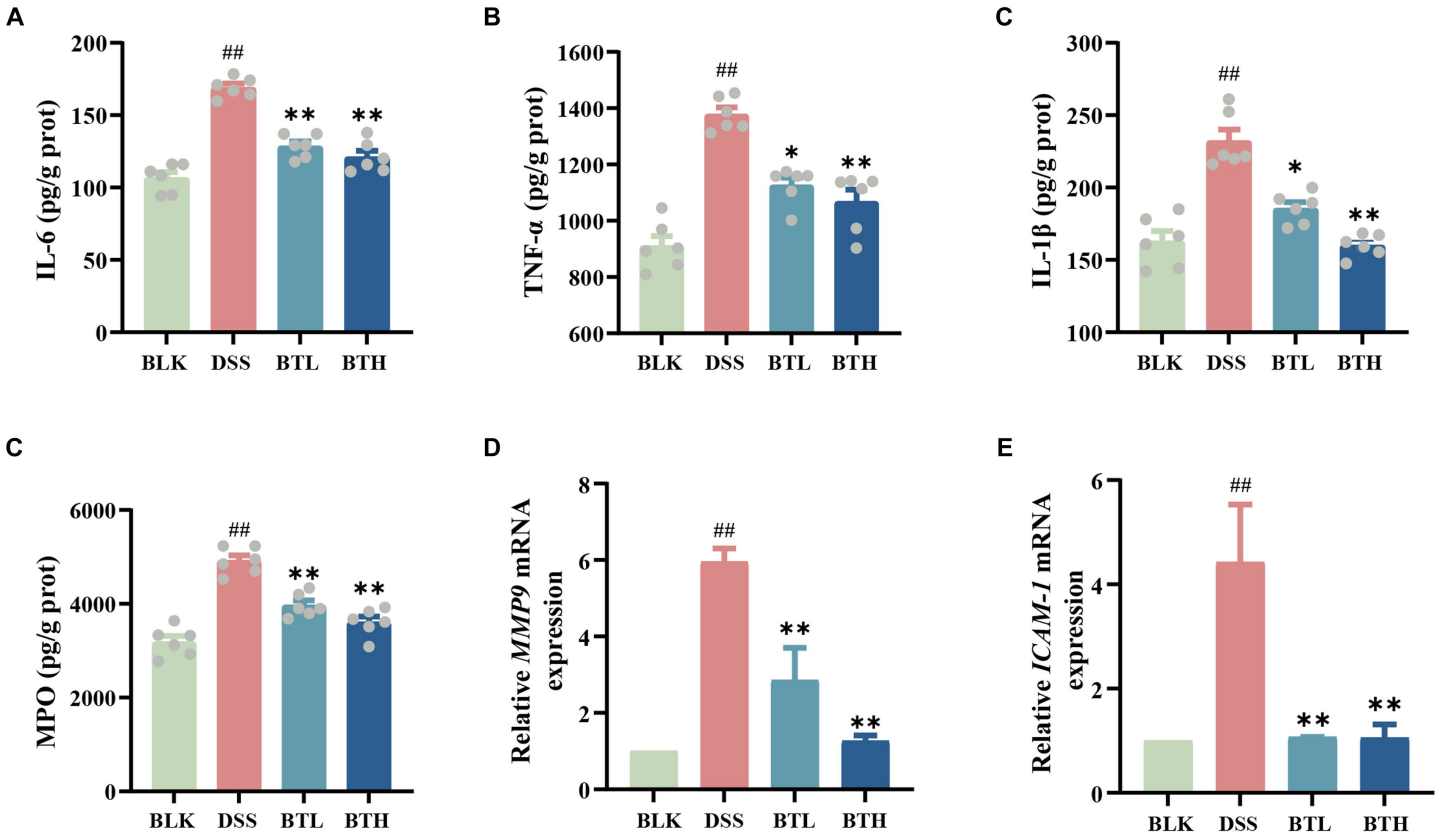
Protective effects of different doses of black tea prevention on the immune barrier of the gut in DSS-induced mouse colitis. **(A–D)** Elisa detection of colitis inflammatory factor protein levels IL-6, TNF-α, IL-β, MPO; **(E,F)** qRT-PCR detection of matrix *metalloproteinase-9* (*MMP9*), and *intercellular adhesion molecule-1* (*ICAM-1*) genes. Data are presented as mean ± SEM (represented by error bars; *n* = 6). The significance of differences between groups was calculated using one-way analysis of variance and Tukey’s multiple comparison test. Compared to the BLK group, ^#^*p* < 0.05, ^##^*p* < 0.01; compared to the DSS group, ^*^p < 0.05, ^**^p < 0.01.

Further ELISA experiments were conducted, and the results demonstrated a significant increase (*p* < 0.05) in various pro-inflammatory cytokines in the colon tissue of DSS-induced mice. In contrast, the black tea treatment group significantly downregulated the expression of pro-inflammatory factors IL-6, TNF-α, and IL-1β in the intestine of DSS-treated mice (Figures 4A–C). MPO levels were quantified through ELISA, and the relative expression levels of *MMP-9* and *ICAM-1* within the colon tissue were evaluated, as depicted in Figures 4D–F. The black tea treatment group suppressed the elevated MPO activity induced by DSS in mice and significantly downregulated the expression of the chemokine *MMP-9* and the adhesion molecule *ICAM-1*, but no significant (*p* > 0.05) difference was observed between the BTH and BTL groups.

### 3.5 Correlation analysis between environmental factors and intestinal microbiota

**Figure 5:**
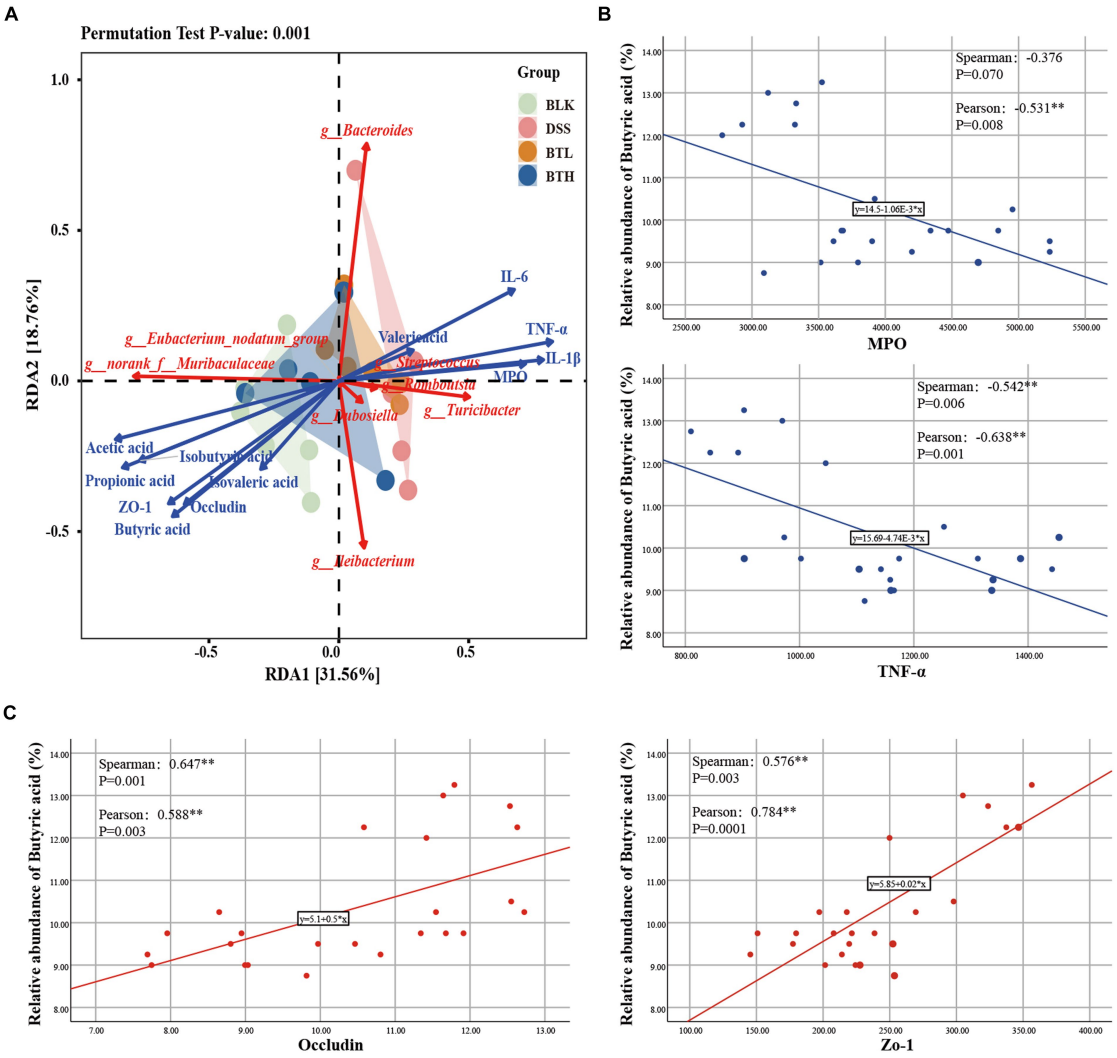
Correlation analysis of intestinal biological barrier, immune barrier and mechanical barrier. **(A)** RDA analysis on fecal gut microbiota with environmental factors. **(B,C)** Correlation analysis of butyric acid with inflammatory factors (MPO, TNF-α) and TJs (Occludin, Zo-1). *p < 0.05, **p < 0.01.

A redundancy analysis (RDA) was subsequently performed, integrating the sample data, genus abundance in gut microbiota, and environmental factors. The red arrows signify the abundance of core microbiota at the genus level, while blue arrows designate trends of various environmental factor members (Figure 5A). Each group of sample points was represented via group-specific ellipses. The explanatory power of the first and second constrained axes of the RDA was 31.56 and 18.76%, respectively. The BLK, BTH, and DSS groups were distinctly separated. Pro-inflammatory cytokines and MPO showed positive correlations with *Romboutsia* and *Bacteroidesc*, but revealed negative correlations with *norank_f_Muribaculacea* and *norank_f_Peptococcacea*. In contrast, TJs and SCFAs exhibited the opposing trend. Furthermore, butanoic acid showed significant negative correlation with MPO and TNF-α, and significant positive correlation with Occludin and ZO-1) (Figures 5B,C). In summation, the findings suggested that the equilibrium of the gut microbiota may be associated with SCFAs, pro-inflammatory factors, and TJs, insinuating that these factors operate in concert to modulate acute colitis.

## 4 Discussion

As shown in Table 1, high concentrations of TF were detected in black tea. The ratio of the aggregate of (TF + TR)/TB was employed as a measure of black tea quality. A higher ratio indicates a more vibrant and sweeter tea infusion. The (TF + TR)/TB ratio for our black tea sample stood at 1.03, indicating a tea of pleasing brightness and superior quality. In addition, the catechin and free amino acid components in the tea leaves were detected, and the results are shown in Supplementary Tables S2, S3.

**Table 1:** Biochemical composition of the sample black tea.

Water extractable	Tea Polyphenols	Free amino acids	Soluble sugar	Soluble protein	Theaflavins (TFs)	Thearubigins (TRs)	Theabrownins (TBs)	Caffeine (CAF)
45.62 ± 0.01	20.30 ± 0.03	3.86 ± 0.01	9.79 ± 0.01	4.00 ± 0.01	0.40 ± 0.03	6.27 ± 0.06	6.45 ± 0.25	2.11% ± 0.02

Characteristic features of colitis induced by DSS primarily include weight loss, rectal bleeding, and complications such as liver and kidney failure; one of the most common potential complications is anemia (30–32). In our study, mice exhibited lower levels of RBC and MCHC, along with higher MCV, which is indicative of severe anemia, likely attributed to excessive rectal bleeding (Figure 1F). Furthermore, DSS-induced mice displayed reduced body weight and decreased food intake, accompanied by significant alterations in colonic mucosal structure due to infiltration of inflammatory cells. These histopathological changes were severe enough to impair normal colonic functions, such as absorption, explaining the weight loss and anemia resulting from excessive rectal bleeding. We found that drinking black tea for 4 weeks in advance alleviated the clinical symptoms of DSS-induced colitis in a dose-dependent manner and had a protective effect on liver and kidney failure, anemia, and other complications. These results are consistent with prior research (23, 33). However, it is worth highlighting that previous studies mainly focused on therapeutic interventions with tea extracts in DSS-induced colitis, while our research investigates the preventive effect of tea extracts when taken as part of the daily diet.

The comparative analysis of species differences between the BTL and DSS groups revealed a significant increase in the relative abundance of *Ruminococcus-1*, *Lachnospiraceae_NK4A136_group*, *Marvinbryantia*, and *UBA1819* in the BTL group (Figure 2E). In the LEFSe analysis, the BTL group exhibited significant enrichment in *Akkermansia* and *Paludicola*. In the BTH group, the genus *Eubacterium_siraeum_group* was dominant. Notably, *Ruminococcus-1* and *Paludicola* both belong to the Ruminococcaceae family, *Lachnospiraceae_NK4A136_group* and *Marvinbryantia* belong to the Lachnospiraceae family, and UBA1819 belongs to the Faecalibacterium. Interestingly, in previous research reports, *Ruminococcus* spp., *Faecalibacterium* spp., and Eubacteria spp. were major producers of butyrate, while Akkermansia spp. produces butyrate and propionate, and acetate is produced by various bacteria such as Lachnospiraceae (34). This suggests that even lower concentrations of black tea may be sufficient to induce beneficial changes in the gut microbiota associated with protective effects against colitis. Although the BTL produced more butyrate, The BTH showed a more significant reversal in the decrease of acetic acid, propionic acid, and butyric acids overall, suggesting that higher doses might be more effective at enhancing the overall production of short-chain fatty acids (SCFAs), thus providing a more comprehensive anti-inflammatory response. These findings highlight the dose-dependent nature of black tea’s effects, suggesting that while both dosages provide benefits, the optimal concentration may depend on the specific SCFAs and gut microbiota dynamics targeted.

SCFAs can enhance the mechanical and chemical barriers of the intestinal tract by upregulating TJs (such as claudin-1 and occludin), intestinal mucins, and Muc-2 (35). We observed that in black tea prevention group, the expression of ZO-1 and occludin was similar to that in the normal group. This suggests that active compounds in black tea, such as tea polyphenols and tea polysaccharides, entered the colon and altered the composition of the gut microbiota, exerting beneficial effects that persisted during DSS-induced colitis. Interestingly, the BTL group showed a significant increase in mucin production associated with *Akkermansia* (36), even surpassing the normal group. This suggests that the low-dose group may demonstrates a more pronounced effect in protect the intestinal chemical barrier by promoting mucin production and increasing the thickness of the mucin layer. In contrast, the BTH group excels in enhancing the expression of TJs to protect the mechanical integrity of the intestine. However, we acknowledge the absence of cell assays and fecal microbial transplant (FMT) experiments as a limitation in establishing a direct link between the protective effects of black tea and gut microbiota changes. These approaches are also crucial for elucidating the specific molecular mechanisms underlying these effects, further bridging the gap between dietary intake and physiological outcomes. This recognition underscores the need for future research to integrate vitro model and FMT, which would enable a more thorough understanding of how dietary factors influence gut health.

Our study found that black tea reduced pro-inflammatory cytokine levels and MPO activity in the intestine, which are indicators of inflammation, suggesting its potential role in intestinal immune modulation. Significantly, butanoic acid demonstrated a notable negative correlation with MPO and TNF-α, (Figure 5B). These findings underline the potential anti-inflammatory role of butanoic acid via the inflammatory responses. Daniela et al. (37) study indicates that SCFAs, particularly butyrate, play a significant role in immunomodulation. Transporters facilitate the uptake of SCFAs, promoting cellular metabolism, while SCFAs may also signal through cell surface G-protein-coupled receptors (GPCRs), activating signaling cascades that control immune functions. Yang et al. (4) research shows that SCFAs can protect the intestines from inflammation by enhancing the production of IL-22.The tea may influence immune cells via microbial metabolites like SCFAs, altering the release of inflammatory agents. Furthermore, we conducted an assessment of two genes, *MMP-9* and *ICAM-1*. The former, by cleaving chemokine precursors, recruits additional effector cells within inflammatory tissues, while the latter upregulates, resulting in increased intestinal epithelial permeability under inflammatory conditions. Importantly, both of these genes are intricately linked with the pathogenesis of inflammatory bowel diseases (38). A substantial body of research has established the association of both genes with the NF-κB signaling pathway, a classical inflammatory cascade considered to be of paramount importance in immune regulation and the orchestration of inflammatory responses in the organism (39). Importantly, cytokines such as IL-6, TNF-α, and IL-1β can activate the NF-κB pathway in the majority of cell types, subsequently resulting in the expression of *MMP-9* and *ICAM-1*. Our research reveals that, in comparison to the DSS group, the black tea intervention group demonstrated a decrease in gene expression for both genes. We propose that this pathway serves as a crucial target through which black tea mitigates DSS-induced acute colitis.

## 5 Conclusion

Based on the results obtained, it is conclude that pretreatment of black tea could increase the beneficial bacteria, at the same time decrease the harmful bacteria in the gut of DSS-induced colitis mice, and also elevate the levels of SCFAs contributing to the suppression of inflammatory factor release, which in turn alleviated the activation of NF-κB signaling pathway, resulting in the reduced expression of *MMP-9* and *ICAM-1*. Such findings indicates that black tea preserves intestinal homeostasis through balancing barriers and microbiota.

## Data availability statement

The datasets presented in this study can be found in online repositories. The names of the repository and accession number can be found at: NCBI: PRJNA1059139.

## Ethics statement

The animal study was approved by the Experimental Animal Ethics Committee of Zhejiang University (ZJU20190004). The study was conducted in accordance with the local legislation and institutional requirements.

## Author contributions

YS: Data curation, Investigation, Writing – original draft. SG: Investigation, Writing – review & editing. JZ: Writing – review & editing. PX: Conceptualization, Funding acquisition, Writing – review & editing. YW: Funding acquisition, Supervision, Writing – review & editing.

## Footnotes

^1^ www.majorbio.com
