# Supplementary material for: Black tea preserves intestinal homeostasis through balancing barriers and microbiota in mice
Source: Front Nutr. 2024 May 21;11:1367047. doi: 10.3389/fnut.2024.1367047 (PMC11148374; doi:10.3389/fnut.2024.1367047)
Supplement: Supplementary file 1 [file Data_Sheet_1.docx]

**Supplementary Materials**

**Table S1:** The sequences of Real-time PCR primers are shown in the following table (5'-3').

| Target gene | Primer sequence (5′-3′) |
| --- | --- |
| *β-actin* | CTACCTCATGAAGATCCTGACC CACAGCTTCTCTTTGATGTCAC |
| *MMP-9* | CGCCACCACAGCCAACTATGAC CTGCTTGCCCAGGAAGACGAAG |
| *Muc-2* | TGCTGACGAGTGGTTGGTGAATG TGATGAGGTGGCAGACAGGAGAC |
| *ICAM-1* | AGTCGTCCGCTTCCGCTACC AGGGTGAGGTCCTTGCCTACTTG |

**Table S2.** Catechin content (%) of black tea catechins in the samples

| Catechin | Content % |
| --- | --- |
| GA | 0.30 ± 0.01 |
| GC | 0.11 ± 0.00 |
| EGC | 0.21 ± 0.01 |
| C | 0.38 ± 0.02 |
| EC | 0.11 ± 0.01 |
| EGCG | 0.11 ± 0.01 |
| GCG | 0.12 ± 0.01 |
| ECG | 0.30 ± 0.03 |
| CG | 0.15 ± 0.00 |

**Table S3.** Free amino acid content of sample black tea (%)

| Free amino acid | Content % |
| --- | --- |
| Asp | 0.05 ± 0.00 |
| Glu | 0.37 ± 0.02 |
| Asn | 0.04 ± 0.00 |
| Ser | 0.11 ± 0.02 |
| Gln | 0.08 ± 0.01 |
| His | 0.02 ± 0.00 |
| Gly | 0.03 ± 0.00 |
| Thr | 0.06 ± 0.00 |
| Arg | 0.05 ± 0.01 |
| Ala | 0.03 ± 0.01 |
| Gaba | 0.03 ± 0.01 |
| Thea | 0.78 ± 0.05 |
| Tyr | 0.06 ± 0.01 |
| Val | 0.03 ± 0.01 |
| Trp | 0.05 ± 0.01 |
| Phe | 0.09 ± 0.01 |
| Ile | 0.08 ± 0.01 |
| Leu | 0.10 ± 0.01 |
| Lys | 0.16 ± 0.01 |

**
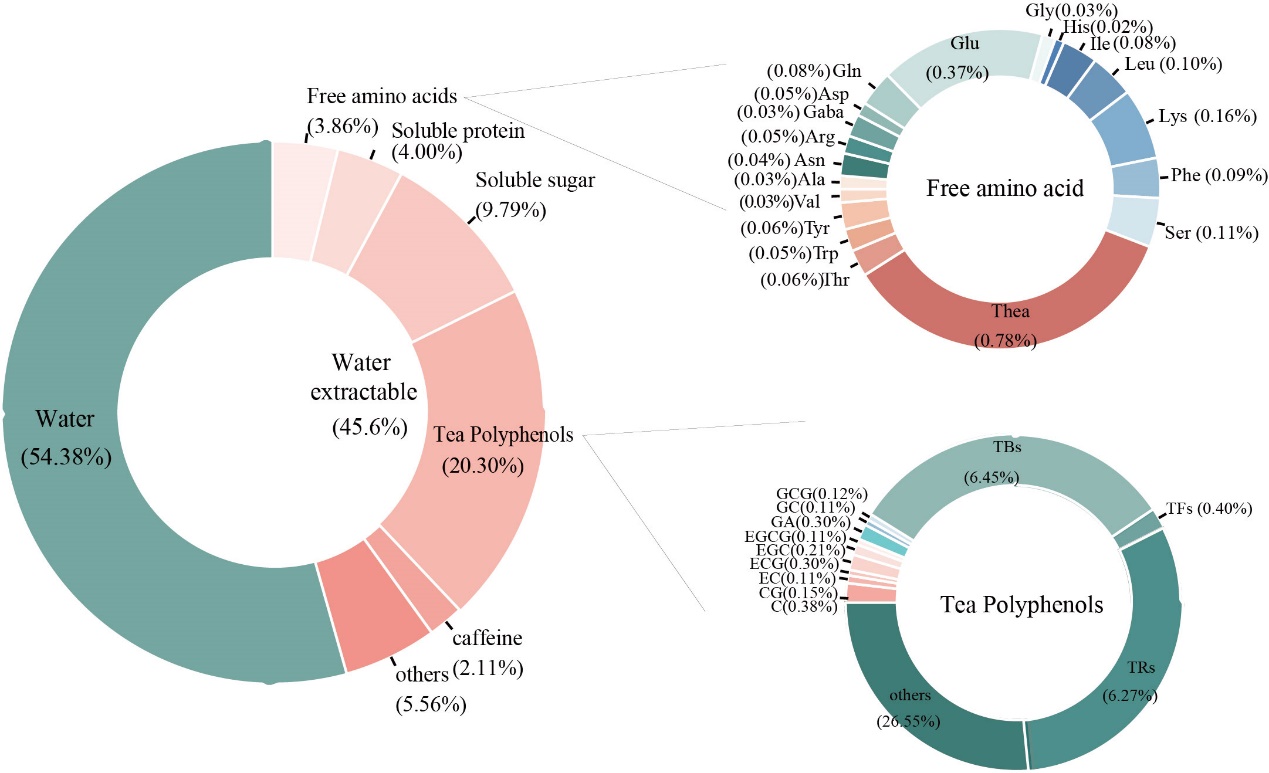
**

**Fig. S1.** Composition ratio of key biochemical constituents in black tea. Representation of biochemical component proportions in black tea using a doughnut chart. (TFs: theaflavins; TRs: thearubigins; TBs: theabrownins)

**
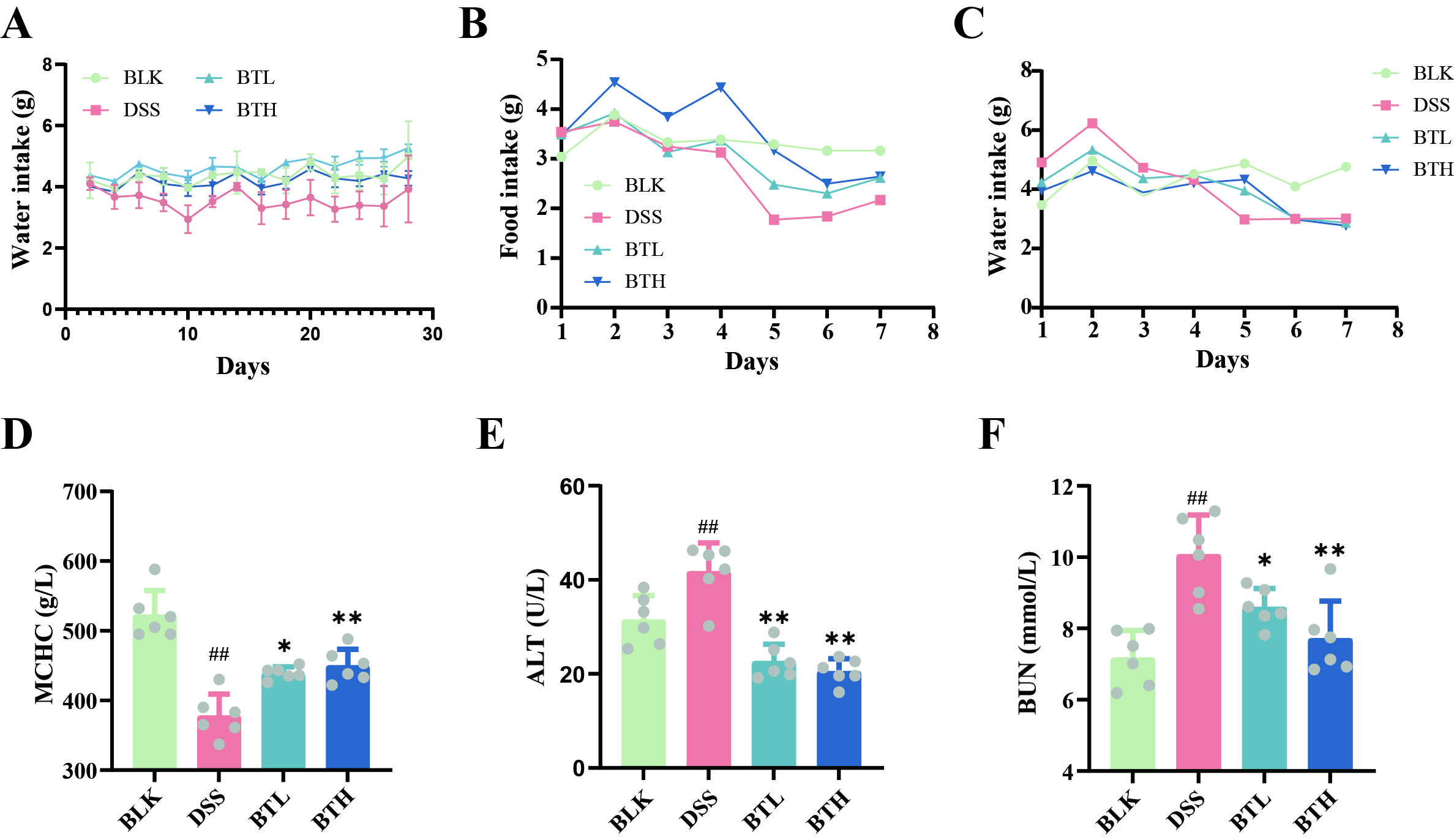
Fig. S2.** (A) Changes in water intake over 4 weeks of tea consumption; (B) Food intake during DSS-induced acute colitis; (C) Water intake during DSS-induced acute colitis; (D) Mean corpuscular hemoglobin concentration (MCHC); (E) Alanine aminotransferase (ALT) activity; (F) Blood urea nitrogen (BUN) levels; Data are presented as mean ± SEM (represented by error bars; n = 6). The significance of differences between groups was calculated using one-way analysis of variance and Tukey's multiple comparison test. Compared to the BLK group, #P < 0.05, ##P < 0.01; compared to the DSS group, *P < 0.05, ** P < 0.01.
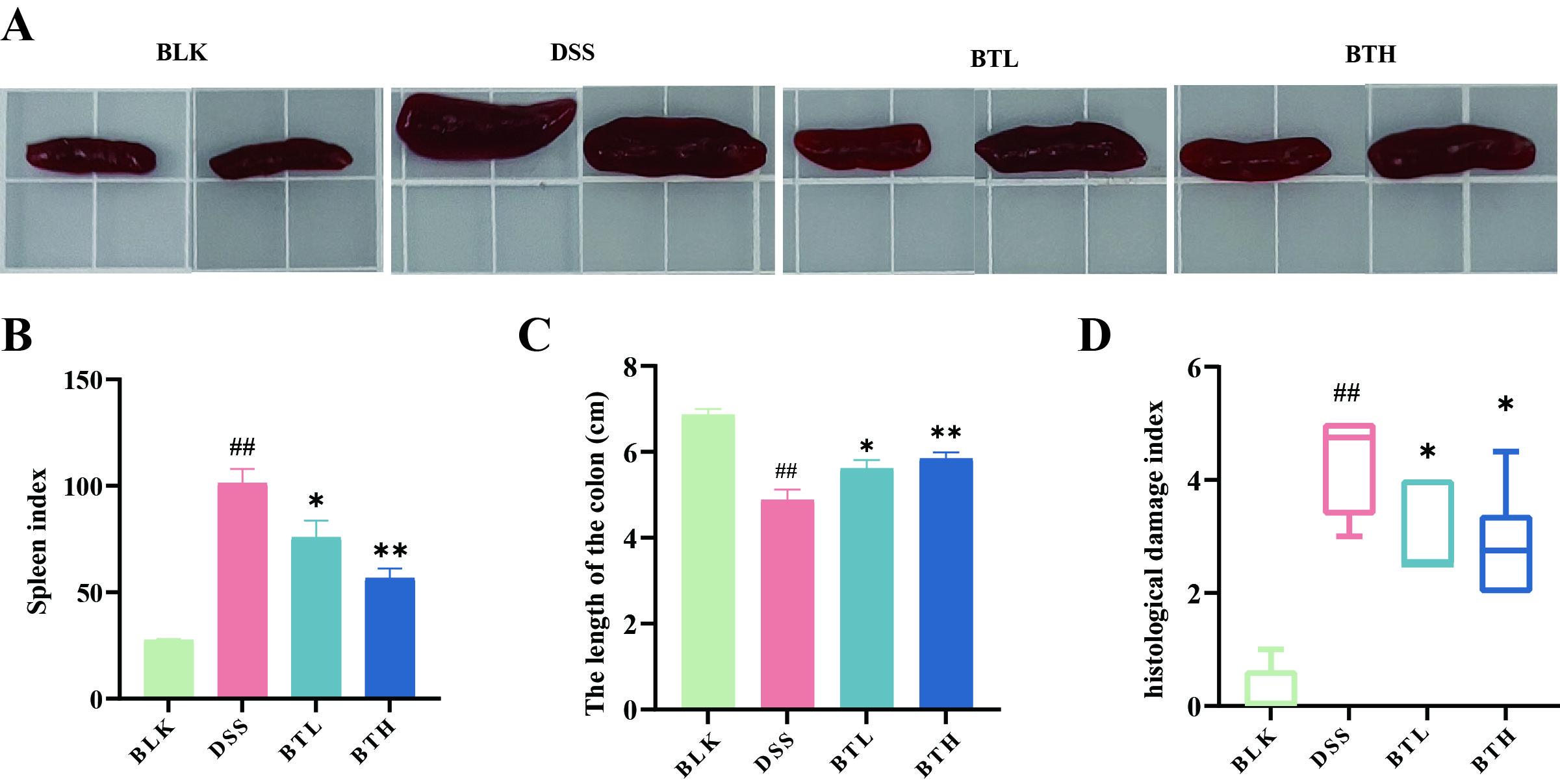
**Fig. S3.** (A) Images of the spleen; (B) Spleen index; (C) Colon length; (D) Histopathological scoring. Data are presented as mean ± SEM (represented by error bars; n = 6). The significance of differences between groups was calculated using one-way analysis of variance and Tukey's multiple comparison test. Compared to the BLK group, #P < 0.05, ##P < 0.01; compared to the DSS group, *P < 0.05, ** P < 0.01.

**
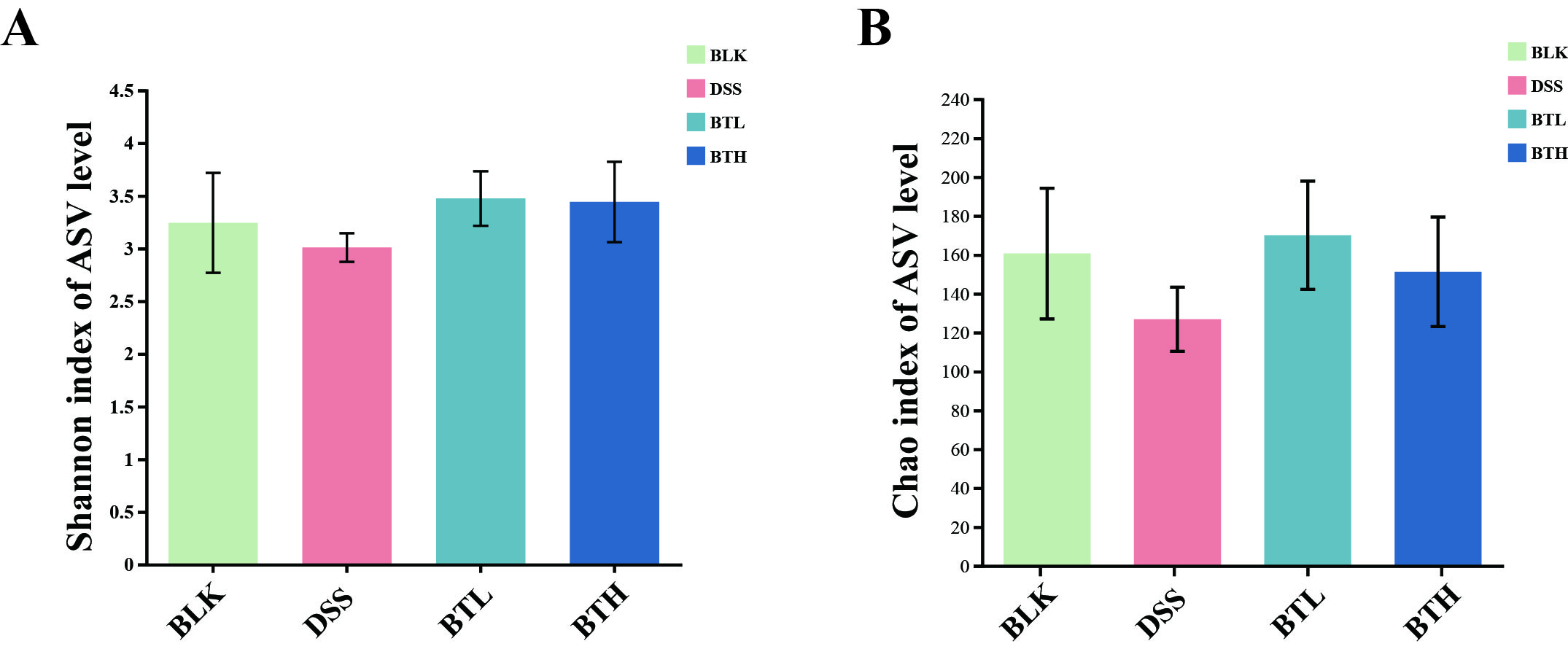
Fig.** **S4.** (A) Alpha diversity analysis using the Shannon index; (B) Alpha diversity analysis using the Chao index.


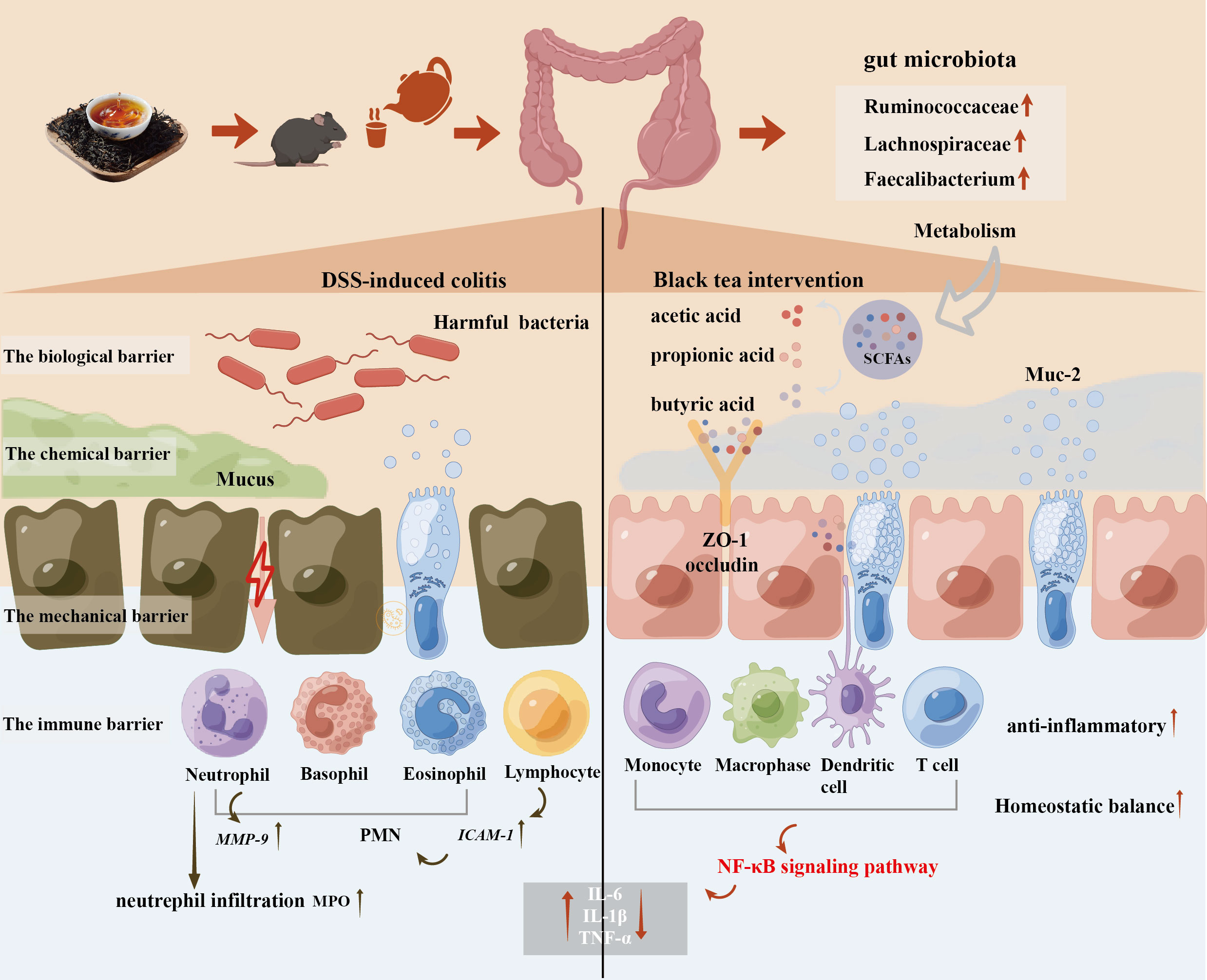


**Fig. S5.** Proposed mechanism by Black tea preserves intestinal homeostasis
